# Supplementary material for: “When in Doubt, Ask the Patient”: A Quantitative, Patient-Oriented Approach to Formative Assessment of CanMEDS Roles
Source: MedEdPORTAL. 2021 Jul 21;17:11169. doi: 10.15766/mep_2374-8265.11169 (PMC8292435; doi:10.15766/mep_2374-8265.11169)
Supplement: Supplementary file 1 — Patient Recruitment.docxOCCAT Version 1.1.docx [file mep_2374-8265.11169-s001.zip › A. Patient Recruitment.docx]

***
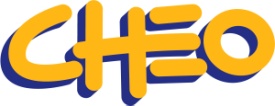

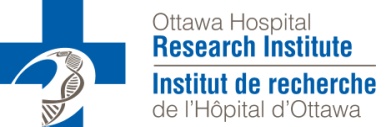

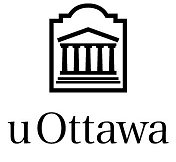

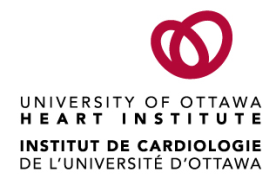
***

**APPENDIX A: PARTICIPANT INFORMED CONSENT FORM**

**Title of Study:** A Patient-Oriented Approach to Formative Assessment of CanMEDS Roles

**Principal Investigator (PI):** Dr. Karen Fung-Kee-Fung, 613-798-5555 ext. 78595

**Co-Investigators:** Dr. Meghan McConnell

Dr. Emanuela Ferretti

Dr. Adam Garber

Ashley Esteves

Participation in this study is voluntary. Please read this Participant Informed Consent Form carefully before you decide if you would like to participate. Ask the study team as many questions as you like. **Your decision to complete and return the questionnaire will be interpreted as an indication of your agreement to participate in the study.**

**Why am I being given this form?**

You are being asked to participate in this research study because you were provided with medical care and/or advice by a clinical fellow during your visit at The Ottawa Hospital's Neonatal-Perinatal Consultation Clinic earlier today.

**Why is this study being done?**

The Ottawa Hospital, in affiliation with the University of Ottawa, is involved in post-graduate medical education (PGME). This means that following graduation from medical school, young doctors are trained here to become specialist physicians. During your visit today, you were assessed by a clinical fellow doing a fellowship in either NPM (Neonatal-Perinatal Medicine) and/or MFM (Maternal-Fetal Medicine). A fellowship is further training that some medical trainees choose to pursue once they successfully complete their residency (training in a medical specialty). At The Ottawa Hospital (TOH) clinical fellows are under the supervision of staff physicians with many years of experience; therefore, the trainee who provided medical care/advice to you earlier today was directly supervised by a staff physician with many years of experience.

Throughout their training, fellows are continually assessed by their supervising physicians; the feedback they get helps them identify areas in which they need improvement. Patients are not traditionally asked to provide feedback, but we believe that they are in a unique position to make valuable judgments about certain aspects of the fellows' behaviour (e.g. how well and clearly they communicate, how patient and compassionate they appear, etc.). In order to engage patients in the assessment of fellows, our research group has developed a questionnaire. The questions of this questionnaire were designed to evaluate the patient's experience with the fellow during the consultation visit; the questionnaire contains questions about how professional the fellow appeared, how well s/he communicated, and how good s/he was at advising you, the patient, on how to lead a healthy lifestyle.

The goal of this study is to help medical fellows become *better* at what they do so that patients have a better experience when they encounter them. We believe that your answers to these questions will help identify what skills the fellows need to improve on.

We estimate that 150 patients and about 12 clinical fellows (MFM & NPM) will participate in this study.

**What is expected of me?**

If you agree to participate in this study, then you will be asked to complete an anonymous questionnaire composed of 29 questions about the clinical fellow who you received medical care and/or advice from. You may skip any questions that make you uncomfortable or that you do not wish to answer. Completing the questionnaire will take approximately 5 minutes.

**How long will I be involved in the study?**

The entire study will last approximately 24 months. Your participation in the study will only involve a single encounter during which you will be asked to complete the 29 item questionnaire. As previously mentioned, the questionnaire will take approximately 5 minutes to complete.

**What are the potential risks I may experience?**

There are no risks associated with participating in this study. The questionnaire is anonymous and any information that you provide will be maintained in full confidentiality. You will not have to answer any questions that make you feel uncomfortable.

Fellows will **NOT** get to review your individual questionnaire; they will, however, be provided with combined results of all of their patients' feedback. Patient feedback will be used for formative purposes only (i.e. to help the trainee become *'better'* in areas in which s/he needs improvement) and **not** to assign a passing or failing grade to the trainee.

**Can I expect to benefit from participating in this research study?**

There is no direct benefit to you from taking part in this study. It is hoped that this study will help the researchers develop and fine-tune an assessment method that will help guide the professional growth of medical trainees. This would ultimately result in better patient care and improved patient experience.

**Do I have to participate? What alternatives do I have?**

Your participation in this study is voluntary. This means that everyone will respect your decision of whether or not you want to be in the study. Your decision will not have any impact on your current or future care at The Ottawa Hospital.

**Will I be paid for my participation or will there be any additional costs to me?**

You will not be paid for your participation in this study, nor will there be any additional costs to you. You will, however, be provided with a paid parking voucher if you'll choose to stay at the hospital slightly longer in order to complete the questionnaire.

**How is my personal information being protected?**

- All information collected during your participation in this study will be collected anonymously. There will be no link between your completed survey and your personal identifying information. Your completed survey will only be linked to the clinical fellow who provided you with medical care and/or advice during your visit to the Ottawa Hospital.
- All study records will be stored securely.
- You will not be identifiable in any publications or presentations resulting from this study.
- For audit purposes only, your original study records may be reviewed under the supervision of Dr. Karen Fung Kee Fung’s staff by representatives from:
  - the Ottawa Health Science Network Research Ethics Board (OHSN-REB),
  - the Ottawa Hospital Research Institute,
  - the Children’s Hospital of Eastern Ontario Research Ethics Board
- Research records will be kept for 10 years, after this time they will be destroyed.

**Who do I contact if I have any further questions or would like to find out about study results?**

If you have any questions about this study or would like to obtain a copy of the study results, please contact Dr. Fung-Kee-Fung at 613-798-5555 ext. 78595 or kfung@toh.on.ca.

The Ottawa Health Science Network Research Ethics Board (OHSN-REB) and the Children’s Hospital of Eastern Ontario’s Research Ethics Board (CHEO-REB) have reviewed the plans for this research study. If you have any questions about your rights as a research participant, you may contact the Chairperson of OHSN-REB at 613-798-5555 ext. 16719 or the Chairperson of CHEO-REB at 613-737-7600 ext. 3272.

***Participation in this study is voluntary. Your decision to complete and return the questionnaire will be interpreted as an indication of your agreement to participate in the study.***
