# Supplementary material for: “When in Doubt, Ask the Patient”: A Quantitative, Patient-Oriented Approach to Formative Assessment of CanMEDS Roles
Source: MedEdPORTAL. 2021 Jul 21;17:11169. doi: 10.15766/mep_2374-8265.11169 (PMC8292435; doi:10.15766/mep_2374-8265.11169)
Supplement: Supplementary file 1 — Patient Recruitment.docxOCCAT Version 1.1.docx [file mep_2374-8265.11169-s001.zip › B. OCCAT Version 1.1.docx]

**Appendix B**

**Ottawa CanMEDS Competency Assessment Tool (OCCAT)**

**Version 1.1**

**PATIENT** **QUESTIONNAIRE**

**Study ID #: _________________________ Date: ___________________________**

**Please rate your interaction with your doctor during your visit today.**

| **CRITERIA**  **COMMUNICATOR** | **Not assessable** | **1 Non-Satisfactory** | **2 Less than satisfactory** | **3 Satisfactory** | **4 Good** | **5 Excellent** |
| --- | --- | --- | --- | --- | --- | --- |
| 1. Addressed you by name |  |  |  |  |  |  |
| 1. Introduced himself/herself |  |  |  |  |  |  |
| 1. Spent the time needed to gather all the details about my condition |  |  |  |  |  |  |
| 1. Listened attentively to me |  |  |  |  |  |  |
| 1. Made me feel comfortable |  |  |  |  |  |  |
| 1. Clearly explained the rationale of the treatment plan |  |  |  |  |  |  |
| 1. Answered all my questions using words I understand |  |  |  |  |  |  |
| 1. Showed compassion even when delivering bad news |  |  |  |  |  |  |
| 1. Asked my opinion about treatment options |  |  |  |  |  |  |
| 1. Explained how my other care givers will be informed |  |  |  |  |  |  |
| 1. Overall how do you rate this fellow as a COMMUNICATOR? |  |  |  |  |  |  |
| **HEALTH ADVOCATE** |  |  |  |  |  |  |
| 1. Asked questions about my lifestyle choices that could influence my health and/or pregnancy outcomes (e.g., Smoking, alcohol consumption, drug use, weight loss) |  |  |  |  |  |  |
| 1. Explained the risks associated with my personal choices |  |  |  |  |  |  |
| 1. Provided options for programs to promote my health (e.g., Quit smoking, dietician, drug rehab) |  |  |  |  |  |  |
| 1. Offered referrals to the other support services related to my needs (e.g., social worker, psychologist, dietician) |  |  |  |  |  |  |
| 1. Advised me of screening programs that can optimize the outcome for me and my baby |  |  |  |  |  |  |
| 1. Explained ways to optimize my baby’s outcome |  |  |  |  |  |  |
| 1. Explained what would happen to my baby after birth (Neonatal Intensive Care Unit or Children’s Hospital of Eastern Ontario) including further consultation and investigation |  |  |  |  |  |  |
| 1. Overall how do you rate this fellow as a HEALTH ADVOCATE? |  |  |  |  |  |  |
| **PROFESSIONAL** |  |  |  |  |  |  |
| 1. Dressed professionally |  |  |  |  |  |  |
| 1. Clean and well groomed |  |  |  |  |  |  |
| 1. Appeared confident |  |  |  |  |  |  |
| 1. Treated me as a person, not just another “case” |  |  |  |  |  |  |
| 1. Was non-judgmental regarding my lifestyle |  |  |  |  |  |  |
| 1. Was non-judgmental regarding my choices for treatment |  |  |  |  |  |  |
| 1. Interacted with me in a respectful, compassionate manner |  |  |  |  |  |  |
| 1. Overall how do you rate this fellow as a PROFESSIONAL? |  |  |  |  |  |  |

**Comments:**

________________________________________________________________________________________________________________________________________________________________________________________________________________________________________________________________________________________________________________________________________________________________________________________________________

**Thank you for your participation. Your opinion is very valuable to us.**

| Please rate the following statements regarding your participation in this process today. | **1 Strongly Disagree** | **2 Disagree** | **3 Neutral** | **4 Agree** | **5 Strongly Agree** |
| --- | --- | --- | --- | --- | --- |
| 1. The patient’s opinion is important when assessing doctors’ skills |  |  |  |  |  |
| 1. I would participate in this process again if given the opportunity? |  |  |  |  |  |

**Ottawa CanMEDS Competency Assessment Tool (OCCAT)**

**Version 1.1**

**SELF-ASSESSMENT** **QUESTIONNAIRE**

**Study ID #: _________________________ Date: ____________________________**

**Please rate your interaction with the patient you have just seen in consultation.**

| **CRITERIA**  **COMMUNICATOR** | **Not assessable** | **1 Non-Satisfactory** | **2 Less than satisfactory** | **3 Satisfactory** | **4 Good** | **5 Excellent** |
| --- | --- | --- | --- | --- | --- | --- |
| 1. Addressed patient by name |  |  |  |  |  |  |
| 1. Introduced myself to patient |  |  |  |  |  |  |
| 1. Spent the time needed to gather all the details about condition |  |  |  |  |  |  |
| 1. Listened attentively to patient |  |  |  |  |  |  |
| 1. Made patient feel comfortable |  |  |  |  |  |  |
| 1. Clearly explained the rationale of the treatment plan |  |  |  |  |  |  |
| 1. Answered all patients’ questions using words they would understand |  |  |  |  |  |  |
| 1. Showed compassion even when delivering bad news |  |  |  |  |  |  |
| 1. Asked patient’s opinion about treatment options |  |  |  |  |  |  |
| 1. I explained how patient’s other care givers will be informed |  |  |  |  |  |  |
| 1. Overall how do you rate yourself as a COMMUNICATOR? |  |  |  |  |  |  |
| **HEALTH ADVOCATE** |  |  |  |  |  |  |
| 1. Asked questions about patient’s lifestyle choices that could influence my health and/or pregnancy outcomes (e.g., Smoking, alcohol consumption, drug use, weight loss) |  |  |  |  |  |  |
| 1. Explained the risks associated with patient’s personal choices |  |  |  |  |  |  |
| 1. Provided options for programs to promote patient’s health (e.g., Quit smoking, dietician, drug rehab) |  |  |  |  |  |  |
| 1. Offered referrals to the other support services related to patient’s needs (e.g., social worker, psychologist, dietician) |  |  |  |  |  |  |
| 1. Advised patient of screening programs that can optimize the outcome for me and my baby |  |  |  |  |  |  |
| 1. I explained ways to optimize baby’s outcome |  |  |  |  |  |  |
| 1. I explained what would happen to patient’s baby after birth (Neonatal Intensive Care Unit or Children’s Hospital of Eastern Ontario) including further consultation and investigation |  |  |  |  |  |  |
| 1. Overall how do you rate yourself as a HEALTH ADVOCATE? |  |  |  |  |  |  |
| **PROFESSIONAL** |  |  |  |  |  |  |
| 1. Dressed professionally |  |  |  |  |  |  |
| 1. Clean and well groomed |  |  |  |  |  |  |
| 1. Appeared confident |  |  |  |  |  |  |
| 1. Treated patient as a person, not just another “case” |  |  |  |  |  |  |
| 1. Was non-judgmental regarding patient’s lifestyle |  |  |  |  |  |  |
| 1. Was non-judgmental regarding patient’s choices for treatment |  |  |  |  |  |  |
| 1. Interacted with patient in a respectful, compassionate manner |  |  |  |  |  |  |
| 1. Overall how do you rate yourself as a PROFESSIONAL? |  |  |  |  |  |  |

**Comments:**

________________________________________________________________________________________________________________________________________________________________________________________________________________________________________________________________________________________________________________________________________________________________________________________________________

**Thank you for your participation. Your opinion is very valuable to us.**

| Please rate the following statements regarding your participation in this process today. | **1 Strongly Disagree** | **2 Disagree** | **3 Neutral** | **4 Agree** | **5 Strongly Agree** |
| --- | --- | --- | --- | --- | --- |
| 1. The patient’s opinion is important when assessing doctors’ skills |  |  |  |  |  |
| 1. I would participate in this process again if given the opportunity? |  |  |  |  |  |
