# Supplementary material for: “When in Doubt, Ask the Patient”: A Quantitative, Patient-Oriented Approach to Formative Assessment of CanMEDS Roles
Source: MedEdPORTAL. 2021 Jul 21;17:11169. doi: 10.15766/mep_2374-8265.11169 (PMC8292435; doi:10.15766/mep_2374-8265.11169)
Supplement: Supplementary file 1 — Patient Recruitment.docxOCCAT Version 1.1.docx [file mep_2374-8265.11169-s001.zip › Educational_Summary_Report_11169.pdf]

MedEdPORTAL® The AAMC Journal of Teaching and Learning Resources

Original Publication Open Access

"When in Doubt, Ask the Patient": A Quantitative, Patient-Oriented Approach to Formative Assessment of CanMEDS Roles

Ashley Esteves, BScH, Meghan McConnell, PhD, Emanuela Ferretti, MD, MSc, Adam Garber, MD, MSc, Karen Fung-Kee-Fung, MD, MHPE*

*Corresponding author: kfung@toh.ca

Abstract

Introduction: Since the introduction of competency-based frameworks into postgraduate medical curricula, educators have struggled to implement robust assessment tools that document the progression of necessary skills. The global movement towards competency-based medical education demands validated assessment tools. Our objective was to provide validity evidence for the Ottawa CanMEDS Competency Assessment Tool (OCCAT), designed to assess clinical performance in the communicator, professional, and health advocate CanMEDS roles. **Methods:** We developed the OCCAT, a 29-item questionnaire informed by specialty-specific Entrustable Professional Activities and consultation with stakeholders, including patients. Our sample included nine neonatal-perinatal medicine and maternal fetal medicine fellows rotating through antenatal high-risk clinics at the Ottawa Hospital. Following 70 unique encounters, the OCCAT was completed by patients and learners. Generalizability theory was used to determine overall reliability of scores. Differences in self and patient ratings were assessed using analyses of variance. **Results:** Generalizability analysis demonstrated that both questionnaires produced reliable scores (G-coefficient > 0.9). Self-scores were significantly lower than patient scores across all competencies, $F(1, 6) = 13.9$, $p = .007$. Variability analysis demonstrated that trainee scores varied across all competencies, suggesting both groups were able to recognize competencies as distinct and discriminate favorable behaviors belonging to each. **Discussion:** Our findings lend support to the movement to integrate self-assessment and patient feedback in formal evaluations for the purpose of enriched learner experiences and improved patient outcomes. We anticipate that the OCCAT will facilitate bridging to competency-based medical education.

Keywords
CanMEDS, Self-Assessment, Multisource Feedback, Competency-Based Medical Education (Competencies, Milestones, EPAs), Assessment, Communication Skills, Feedback, Neonatal-Perinatal Medicine

Educational Objectives

By using this assessment tool, facilitators will be able to:

1. Evaluate learners' performance of professional, health advocate, and communicator CanMEDS competencies following patient encounters.
2. Engage patients in real-time assessment of postgraduate medical trainees and promote self-reflection among learners.
3. Compare learner scores with patient scores to identify sources of incongruity in order to improve delivery of patient care.

Citation:
Esteves A, McConnell M, Ferretti E, Garber A, Fung-Kee-Fung K. "When in doubt, ask the patient": a quantitative, patient-oriented approach to formative assessment of CanMEDS roles. *MedEdPORTAL*. 2021;17:11169.
https://doi.org/10.15766/mep_2374-8265.11169

Introduction

The CanMEDS Physician Competency Framework (CanMEDS) describes the knowledge, skills, and abilities all physicians need to effectively care for their patients.[1] CanMEDS is currently applied across five continents, making it the most widely recognized physician competency framework. This framework is built on seven distinct yet intertwined roles: medical expert, communicator, collaborator, leader, health advocate, scholar, and professional.[2] Postgraduate training programs throughout Canada strive to produce physicians with the comprehensive range of skills articulated in the CanMEDS framework. The implementation of competency-based frameworks has emphasized the importance of robust, multifaceted assessment systems that enable educators to document the progression of competence in trainees.[3] Such assessment systems should be continuous, comprehensive, and performed in the context of the clinical workplace.[3] The development of such assessment

Copyright © 2021 Esteves et al. This is an open-access publication distributed under the terms of the Creative Commons Attribution license.

systems is not an easy feat. Program directors and educators have struggled to develop and implement assessment tools that document learner progression. Furthermore, within Canada, program directors have indicated that there is a lack of robust, high-quality assessment tools to evaluate performance of the CanMEDS roles, apart from medical expert.[4-7]

Traditionally, assessment of trainees has typically been performed by a supervising health care professional, such as a staff physician. This approach, although perhaps expedient, solicits feedback from only one segment of the interprofessional team to determine whether candidates have reached the benchmarks for clinical performance in the CanMEDS domains. However, faculty evaluators are often not present during many of the postgraduates' clinical encounters, and observations made by other members of the health-care team can provide valuable information to help guide the professional growth of trainees.[8] Patients regularly engage in one-on-one interactions with physician learners. Feedback from patients might provide learners with valuable insight regarding their clinical performance. The end users of health care, patients, are seldom solicited for their opinion, yet they are often in a unique position to make valuable judgments about the physician learners' behavior.

Feedback solicited from more than one source is defined as multisource feedback (MSF) and has been identified as a legitimate tool for assessing CanMEDS competencies.[9] Reports from the medical education literature have demonstrated that performance feedback from multiple sources, including patients, can be used to inform changes in practice.[10,11] In fact, a recent systematic review of MSF found that using patients as assessors was a feasible, reliable, and valid practice.[12] Additionally, researchers have argued that self-assessment is also a useful component of MSF.[13] Self-assessment involves "interpreting data about one's own performance and comparing it against an implicit standard."[14] Self-assessment encourages learners to reflect on their own performance and can help trainees develop metacognitive skills, enhance their learning strategies, and become independent and confident learners.[14] That being said, the accuracy of self-assessment has been highly criticized in the literature because individuals tend to overestimate their performance when compared to a more objective measure.[15] Despite criticisms regarding the accuracy of self-assessment, researchers have shown that it is associated with increased learner motivation and the pursuit of higher goals.[16] In this way, self-assessment promotes a metacognitive approach to learning by involving learners directly in assessment practices.[17]

Our primary objective was to determine the validity of the Ottawa CanMEDS Competency Assessment Tool (OCCAT), a questionnaire designed to assess clinical performance of three intrinsic CanMEDS competencies (professional, communicator, and health advocate) by maternal fetal medicine (MFM) and neonatal-perinatal medicine (NPM) fellows, using Kane's validity framework.[18] From this, we planned to implement the questionnaire as a standard assessment tool for our program. The non–medical expert competencies chosen represented skills universally applicable and valuable to all fields of medicine. While the validation of a novel CanMEDS assessment tool has been documented before,[19-21] our group uniquely investigated performance of intrinsic CanMEDS roles as perceived by patients as well as by self-assessment.

Methods

Setting

Our investigation took place within the antenatal high-risk clinic at the Ottawa Hospital—General Campus in Ontario, Canada, from April 2016 to June 2018. This weekly clinic provided consultation services to pregnant women at high risk of having adverse fetal and/or neonatal outcomes. Approximately 200 clinic visits were recorded annually, with an average of four new patients seen for consultation every week.

Subjects

MFM and NPM subspecialty training programs at the University of Ottawa were 2-year credentialed programs accredited by the Royal College of Physicians and Surgeons of Canada (RCPSC). Residents and fellows performed the consultation for all new patients attending the antenatal high-risk clinic. These experiences often involved education on maternal and fetal risks, communication of management plans to minimize risk, and health advocacy in a sensitive, patient-focused manner. This population was also easily accessible to the principal investigators.

MFM and NPM trainees across all years were approached by a research assistant to participate. Those who did not wish to participate were excluded. It was emphasized to the trainees that their participation was completely voluntary and that their decision to participate (or not) would have no influence on their formal evaluations. Trainees were provided with an informed consent form (Appendix A) and were offered a five-dollar gift card to a coffee shop of their choice for every questionnaire they completed.

All patients over the age of 18 who presented to the antenatal high-risk clinic for an initial consultation during the 26-month investigation period were approached by the research assistant

Copyright © 2021 Esteves et al. This is an open-access publication distributed under the terms of the Creative Commons Attribution license.

to participate. Returning patients and/or those under 18 years of age were excluded. The research assistant approached eligible patients either before or after their scheduled consultation visit. Interested patients were provided with an informed consent form (Appendix A) and the OCCAT (Appendix B) following the completion of their consultation. Patients' participation was incentivized by offering a paid parking voucher. Patient demographic information was not collected.

Completed questionnaires were collected and scores were translated to a secure digital format by the research assistant using deidentified codes. Research ethics board approval to conduct this investigation was obtained from the Ottawa Health Science Network Research Ethics Board (protocol 20150518-01H).

Instrument Design/Scoring Inference

We devised the OCCAT over several months. Conceptualization began with consultation with key stakeholders, including patients, learners, clinic nurses, faculty physicians, and the MFM program coordinator. Stakeholders were voluntarily recruited to join a development group of 10 individuals, two of whom were patients. Group members were presented with the CanMEDS 2015 framework and the following guiding principles to inform choices for item creation: provision of a formative scaffold that would identify perceived strengths and weaknesses in patient interactions within these intrinsic domains, help trainees identify their own developmental and learning needs for effective practice as related to these intrinsic competencies, foster insight among residents into their professional behaviors, allow a gap analysis between how residents perceive themselves and how others perceive them, and promote a shared understanding of what matters most to patients. From these discussions, a prototype tool of 12 items reflecting intrinsic CanMEDS competencies was devised.

Working with the MFM program coordinator, we then disaggregated several of the original items to ensure items were as specific as possible. For example, the communicator items "Addressed you by name" and "Introduced himself/herself" were originally one item. Another original item, "Sits down during the consultation," was reconceptualized as "Listened attentively to me" and "Made me feel comfortable." Originally, the health advocate competency was captured by only one item, "Outlined ways of optimizing my health and my baby's health." We then expanded this item to capture the seven more relevant health advocacy behaviors published in the OCCAT. The wording of three items was changed to remove terminology that required previous medical knowledge. For instance, under the health advocate competency, "Advised me of screening programs that can optimize the outcome of my pregnancy—IPS Screening" was changed to "Advised me of screening programs that can optimize the outcome for me and my baby." The item "Explained ways to optimize my health and pregnancy outcome (e.g., Folic Acid, Vit D, etc.)" was revised to "Explained ways to optimize my baby's outcome." Finally, the item "Stressed the importance of attending all prenatal appointments" was replaced with "Explained what would happen to my baby after birth (Neonatal Intensive Care Unit or Children's Hospital of Eastern Ontario) including further consultation and investigation" to make the item more relevant. The professional competency items were unchanged, apart from adding the "Dressed professionally" item. We also added summary items to each competency asking the rater to assign an overall competency score.

During the study period, competency-based training was introduced by the RCPSC, and Entrustable Professional Activities (EPAs) as key tasks for each discipline were articulated for the NPM programs.[22] As a result, the questionnaire was further revised to reflect these elements of competency-based medical education. For example, the NPM Foundations EPA 7 described the achievement of shared decision-making with families.[22] Milestones within this EPA such as "Use communication skills and strategies that help the family make informed decisions" and "Recognize when strong emotions (such as anger, fear, anxiety, or sadness) are impacting an interaction and respond appropriately" corresponded to items 6 and 8, respectively, within the communicator role. The NPM Core EPA 2 described the achievement of providing antenatal consultations for patients with complex conditions and included milestones such as "Assess a patient's need for additional health services or resources," which corresponded to item 4 in the health advocate role, and "Work with the patient's family to establish goals of care," which corresponded to item 9 in the communicator role.

The questionnaire items were finally vetted by NPM and MFM fellows who were provided with the following questions: Do these items accurately reflect the behaviors that are valued by you and are considered appropriate? Would you add/delete any items? Are any items redundant? Is there any ambiguity in the items? Would you reclassify any behaviors under different CanMEDS roles? Which items could you classify as "least important"? Should there be any open-ended items on the questionnaire that reflect behaviors that are not reflected in the rating items? We also reviewed response options and decided to use a five-point semantic-coded scale (1 = *poor,* 5 = *excellent*) to rate responses, with the option to choose "Not Assessable." Formal beta testing

Copyright © 2021 Esteves et al. This is an open-access publication distributed under the terms of the Creative Commons Attribution license.

of target groups was not performed as the sample size of the learner group was limited.

OCCAT items referred to assessment of the patient's interaction experience with the trainee during the consultation visit alone and did not address the organization of the clinic or service. The final version of the OCCAT consisted of a total of 29 items, with 11 items aligned to the communicator role, eight items aligned to both the health advocate and the professional roles, and two additional items evaluating the perceived importance of the patient opinion in physician assessment and determining whether participants would participate again, given the opportunity. We first developed the OCCAT from the patient perspective, but when put into first-person narrative, it served as a tool for trainees to perform self-assessment (Appendix B). Additional aids including guides and/or manuals to accompany the questionnaire were deemed unnecessary since the tool was designed to be user-friendly and targeted to the general population and verbal instruction was provided by the research assistant.

Generalization Inference

Evidence for generalizability was assessed in two ways. First, we examined whether the OCCAT provided reliable (e.g., reproducible) scores.[23-25] Generalization evidence for our tool was assessed using generalizability theory (G-theory). Originally developed as a way to determine the amount of error involved in the measurement process, G-theory has been used to evaluate generalization evidence in medical education contexts.[26] A strength of using G-theory as a measure of reliability is its ability to identify which factors (e.g., trainee, encounter, competency) contribute to variability in scores. The object of measurement was trainee (t), and encounter was nested within trainee (e:t). A random effects model was used to quantify the sources of variation associated with encounter (e:t) and competency (c). Competency scores were collapsed across items such that mean communicator, health advocate, and professional scores were analyzed. Separate generalizability analyses were conducted for self and patient ratings.

Second, we computed item-total correlations (ITCs) in order to determine whether individual items within the tool were related to the overall construct.[23-25] ITCs enabled us to identify items that were weakly or negatively correlated with average competency scores. That is, ITCs helped to identify items that were behaving differently, were presumed not to measure the same construct, and could be ultimately discarded from the tool. In addition, the internal consistency of items within each competency was estimated using Cronbach's alpha. Individual items were collapsed across encounters such that the mean score for each item was analyzed. This multidimensional analysis was used to determine internal consistency reliability to support Kane's generalization inference that a total score reflects performance.

Extrapolation Inference

Extrapolation validity is supported when assessment scores relate to real-life performance.[25] We were interested in whether differences between trainee self-rating patterns and patient-rating patterns would reflect response processes in real life. We hypothesized that self-assessment scores would be decreased in comparison to patient scores, across all competencies, reflecting a well-documented propensity for patients to rate their physicians leniently.[12,27-29] For each competency and their individual items, we tabulated the mean score and standard error across encounters. More specifically, we took the average score for items relating to communicator ($n = 11$), health advocate ($n = 8$), and professional ($n = 8$) performed for both self-reported scores and patient-reported scores. Next, a 2 (rater: self vs. patient) $\times$ 3 (CanMEDS role) within-subjects analysis of variance (ANOVA) was conducted. In the context of medical education, tools that can discriminate between learners by level of study often contribute to extrapolation evidence.[25] Effect sizes were calculated using partial eta-squared (η^2) for ANOVAs and Cohen *d* for *t* tests. The magnitude of these effect sizes was interpreted using classifications proposed by Cohen[30]: small effect sizes $\approx \eta^2 < 0.02$ and Cohen $d < 0.2$, medium effect sizes $\approx 0.02 < \eta^2 < 0.13$ and $0.2 < d < 0.8$, and large effect sizes $\approx \eta^2 > 0.14$ and $d > 0.8$.

Results

Descriptive Statistics

A total of 69 patients and nine fellows participated in the present study. Collectively, 69 patient questionnaires and 70 fellow questionnaires were completed, with a response rate of 99% and 100%, respectively. Of the nine fellows, seven were NPM fellows, and two were MFM fellows. Questionnaires from three junior fellows and nine senior fellows, including those who had previously participated in their PGY 6 year, were analyzed. On average, 7.7 questionnaires were completed per fellow. The complete set of item means and standard deviations is listed in Table 1.

Scoring Inference

As a measure of scoring inference, we examined the proportion of "Not Assessable" responses. A high proportion of "Not Assessable" responses would suggest that certain items might not be applicable in our assessment contexts. The health advocate competency had the highest proportion of "Not

Copyright © 2021 Esteves et al. This is an open-access publication distributed under the terms of the Creative Commons Attribution license.

Table 1. Mean Patient-Rated (*N* = 69) and Self-Rated (*N* = 70) Scores Across Competency Items and Totals With Associated Standard Distribution and Error

	Patient			Self		
**Role and Item**	***M*[a]**	***SD***	***SEM***	***M*[a]**	***SD***	***SEM***
Communicator	4.6	.22	.027	3.9	.59	.070
Addressed patient by name.	4.4	.43		4.0	.58	
Introduced self to the patient.	4.7	.34		4.1	.71	
Spent the time needed to gather all the details about condition.	4.6	.32		4.1	.73	
Listened attentively to patient.	4.7	.28		4.1	.69	
Made patient feel comfortable.	4.7	.21		4.0	.58	
Clearly explained the rationale of the treatment plan.	4.6	.22		3.9	.69	
Answered all patient's questions using words they would understand.	4.7	.23		3.8	.77	
Showed compassion even when delivering bad news.	4.6	.32		3.9	.47	
Asked patient's opinion about treatment options.	4.2	.39		3.6	.47	
Explained how patient's other caregivers will be informed.	4.5	.17		3.7	.51	
Health advocate	4.5	.33	.041	3.6	.54	.064
Asked questions about patient's lifestyle choices that could influence health and/or pregnancy outcomes (e.g., smoking, alcohol consumption, drug use, weight loss).	4.4	.31		3.3	.84	
Explained the risks associated with patient's personal choices.	4.2	.61		3.6	.67	
Provided options for programs to promote patient's health (e.g., quit smoking, dietician, drug rehab).	3.9	.40		3.4	.79	
Offered referrals to the other support services related to patient's needs (e.g., social worker, psychologist, dietician).	4.2	.48		3.6	.62	
Advised patient of screening programs that can optimize the outcome for patient and patient's baby.	4.4	.39		3.0	.57	
Explained ways to optimize baby's outcome.	4.4	.38		3.8	.62	
Explained what would happen to patient's baby after birth (NICU or CHEO care), including further consultation and investigation.	4.8	.13		3.9	.81	
Professional	4.7	.24	.029	4.1	.71	.085
Dressed professionally.	4.7	.26		4.1	.77	
Clean and well-groomed.	4.7	.30		4.0	.67	
Appeared confident.	4.5	.51		3.9	.57	
Treated patient as a person, not just another "case."	4.8	.21		4.1	.73	
Was nonjudgmental regarding patient's lifestyle.	4.8	.22		4.1	.68	
Was nonjudgmental regarding patient's choices for treatment.	4.8	.24		4.1	.72	
Interacted with patient in a respectful, compassionate manner.	4.9	.17		4.0	.88	

Abbreviations: CHEO, Children's Hospital of Eastern Ontario; NICU, neonatal intensive care unit; *SEM,* standard error of the mean.
[a]Rated on a 5-point scale (1 = *poor*, 5 = *excellent*).

Assessable" responses for both patient and self-assessment questionnaires. More specifically, of a total eight items, six items were rated as "Not Assessable" more than 30% of the time for the patient questionnaires (health advocacy items 1-6), and four items were rated as "Not Assessable" more than 30% of the time for self-assessments (items 2-5). None of the items corresponding to communicator and professional competencies had "Not Assessable" response rates greater than 30%.

Generalization Inference

We applied generalizability theory to determine the internal consistency and reproducibility (e.g., interrater reliability) of our questionnaire. Sources of variance, identified using G-theory, are displayed in Table 2. While we found 0% variance attributable to differences among our trainees, the interaction between trainee and competency (facet t*c) accounted for a large portion of variance across both self (89%) and patient (93%) questionnaires. This finding indicates that both trainees and patients were able to discriminate residents' performance across the three competencies. The variance components were then used to determine the overall reliability of both the patient and self-questionnaires (G-coefficient = 0.926 and 0.928, respectively), which exceeded the recommended standard (Table 2).

We calculated ITCs to identify items that correlated poorly with average competency scores (Table 3). ITCs less than 0.2 suggest that an item may not be measuring the same construct as the others. Only one item, "Explained ways to optimize my baby's outcome," within the health advocacy competency in the patient questionnaire had an ITC below the threshold of 0.2. Low ITCs may result from the propensity of patients to globally assign low ratings. While the mean patient score for this item was only moderately low at 4.4 (*SD* = .38), 36% of patients had opted to respond with "Not Assessable." Meanwhile, this item was found to have an ITC of 0.9 in the mirrored self-questionnaire, suggesting learners had a better understanding of this competency and how the chosen items related to it, as

Copyright © 2021 Esteves et al. This is an open-access publication distributed under the terms of the Creative Commons Attribution license.

Table 2. Analysis of Tool Reliability and Sources of Variability Using G-Theory

	Patient[a]		Self[b]		
Facet	Variance	% Variance	Variance	% Variance	Interpretation
t	0	0	0	0	Variance due to differences between trainees
e:t	0	0	0	0	Variance due to differences across encounters nested within trainee
c	0	0	0.13	4.41	Variance due to differences across competencies
t*c	2.52	92.59	2.61	88.66	Variance due to the interaction between trainees and competencies
e:t*c	0.20	7.41	0.20	6.92	Variance due to the trainees, time, items, plus random error

Abbreviations: c, competency; e, encounter; t, trainee.
[a]G-coefficient = 0.926.
[b]G-coefficient = 0.928.

would be expected. Thus, this item remained in the original questionnaire.

Lastly, we used Cronbach's alpha to analyze the internal consistency of items within each CanMEDS competency. Cronbach's alpha represents the expected correlation between any number of items belonging to a proposed construct.[31] Table 3 displays the Cronbach's alpha for each competency assessed by the questionnaires. The internal consistency of items within a competency ranged from .75 to .99. We noted that the lowest values among patients and learners originated from the health advocate competency.

Extrapolation Inference

To find evidence for extrapolation validity, our group assessed how the OCCAT could relate to real-life outcomes. To begin, we compared rating trends of patients and learners. Mean scores from both questionnaires with their associated standard errors

Table 3. Analysis of Individual Intrinsic Item Behaviors and Internal Reliability

	Patient		Self	
Role and Item	ITC	Cronbach's alpha	ITC	Cronbach's alpha
Communicator				
Addressed patient by name.	0.9	.91	0.9	.98
Introduced self to the patient.	0.6		1.0	
Spent the time needed to gather all the details about condition.	0.8		0.9	
Listened attentively to patient.	0.9		1.0	
Made patient feel comfortable.	0.9		1.0	
Clearly explained the rationale of the treatment plan.	0.9		1.0	
Answered all patient's questions using words they would understand.	0.7		1.0	
Showed compassion even when delivering bad news.	0.3		0.9	
Asked patient's opinion about treatment options.	0.8		0.7	
Explained how patient's other caregivers will be informed.	0.3		0.8	
Health advocate				
Asked questions about patient's lifestyle choices that could influence health and/or pregnancy outcomes (e.g., smoking, alcohol consumption, drug use, weight loss).	0.9	.75	0.6	.88
Explained the risks associated with patient's personal choices.	0.4		0.8	
Provided options for programs to promote patient's health (e.g., quit smoking, dietician, drug rehab).	0.8		0.8	
Offered referrals to the other support services related to patient's needs (e.g., social worker, psychologist, dietician).	0.6		0.8	
Advised patient of screening programs that can optimize the outcome for patient and patient's baby.	0.2		0.3	
Explained ways to optimize baby's outcome.	0.1		0.9	
Explained what would happen to patient's baby after birth (NICU or CHEO care), including further consultation and investigation.	0.8		0.5	
Professional				
Dressed professionally.	0.8	.89	1.0	.99
Clean and well-groomed.	0.8		1.0	
Appeared confident.	0.8		0.9	
Treated patient as a person, not just another "case."	0.8		1.0	
Was nonjudgmental regarding patient's lifestyle.	0.5		1.0	
Was nonjudgmental regarding patient's choices for treatment.	0.7		1.0	
Interacted with patient in a respectful, compassionate manner.	0.8		0.9	

Abbreviations: CHEO, Children's Hospital of Eastern Ontario, ITC, item-total correlation; NICU, neonatal intensive care unit.

Copyright © 2021 Esteves et al. This is an open-access publication distributed under the terms of the Creative Commons Attribution license.

are visualized in the Figure. There was a main effect for rater, $F(1, 8) = 13.2$, $p = .007$, $\eta^2 = 0.62$, demonstrating that trainee performance was rated more favorably by patients compared to self-assessment ($M = 3.9$ [95% CI, 3.4-4.3] and 4.6 [95% CI, 4.4-4.7], respectively). There was also a main effect of CanMEDS roles, $F(2, 16) = 7.71$, $p = .005$, $\eta^2 = 0.49$. Subsequent *t* tests revealed that mean health advocate scores ($M = 4.1$ [95% CI, 3.8-4.3]) were significantly lower than mean professional scores ($M = 4.4$ [95% CI, 4.1-4.7], $p_{bonferroni} = .002$, Cohen $d = 1.17$). Mean communicator scores ($M = 4.2$ [95% CI, 4.0-4.5]), however, did not differ from either health advocate or professional scores. There was no interaction between rater and CanMEDS role.

The final item on the OCCAT addressed the importance of patient opinions when assessing physician skills. The mean score from the trainee questionnaires was 4.3, and the mean score from the patient questionnaires was 4.7. While patients' responses indicated that their involvement in medical education was more important compared to the trainees' responses, both groups highly valued patient evaluation.

Discussion

We developed the OCCAT to assess postgraduate subspecialty learner competency across three of the non–medical expert CanMEDS roles and collected validity evidence according to Kane's framework.[18] The roles assessed by our questionnaire were communicator, health advocate, and professional. We analyzed 69 patient questionnaires and 70 self-questionnaires reflecting 70 unique encounters across nine learners.

Our steps toward tool development lend evidence for Kane's scoring inference since items were informed by empirically established EPAs[22] and patient consultation to ensure key aspects of the communicator, health advocate, and professional roles were captured. We carefully reviewed the wording of items to ensure they could be easily understood by the lay population. We decided on a 5-point semantic-coded scale (1 = *poor,* 5 = *excellent*) to rate responses, with the option to choose "Not Assessable." ITCs were computed to identify items that were presumed not to measure the same construct and could ultimately be discarded from the tool. No items exhibited an ITC less than 0.2 across both questionnaires. Thus, we decided all items would remain within their original competencies.

The OCCAT produced reliable scores, with G-coefficients exceeding the recommended threshold of 0.9 for high-stakes assessments, thereby providing evidence for generalization validity.[23-25] Demonstrating the internal consistency of a tool also provides evidence for the generalization inference.[25] Items across both groups and all competencies demonstrated satisfactory internal consistency greater than 0.7. While some have argued this threshold should be 0.9 for clinical applications,[31] the range of acceptable alphas remains wide.[32] We recognize that our small trainee sample size and lack of trainee variability as reported in Table 2 may limit generalization

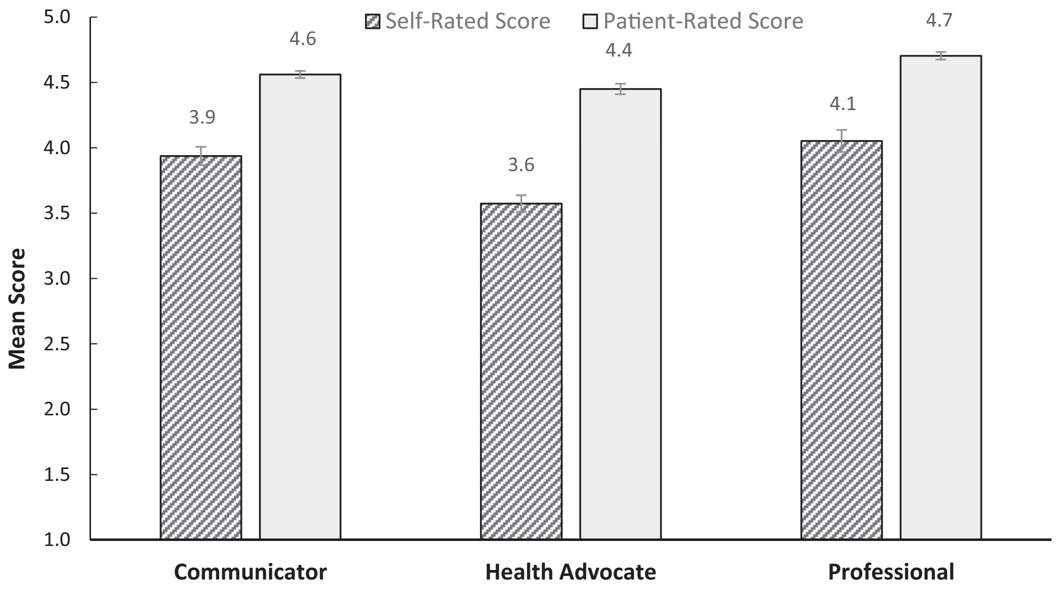

Figure. Comparison of mean patient-rated and self-rated competency scores with associated standard error. Rated on a 5-point semantic-coded scale (1 = *poor*, 5 = *excellent*).

Copyright © 2021 Esteves et al. This is an open-access publication distributed under the terms of the Creative Commons Attribution license.

evidence.[25] We suspect that the lack of trainee variability may be related to the timing of our investigation. Data were collected beginning in April to avoid the expected adjustment period of starting fellowship in July. However, we are optimistic about the finding that trainees demonstrated high variability in scoring themselves across the three competencies. Interestingly, the majority of score variance (93%) originated from the interaction between trainee and competency. This is significant to the field of medical education because it demonstrates that both patients and trainees can recognize the competencies as distinct and discriminate favorable behaviors belonging to each, rather than having their ratings reflect a halo effect. To our knowledge, there is no documentation in the literature describing patient capacity to discriminate between physician competencies. However, since the tool was created with this objective in mind and items were carefully worded for the lay public, these data suggest that patients can provide important insight into different intrinsic competencies. Our findings also revealed that mean health advocate scores were significantly lower than mean professional scores, while mean communicator scores did not differ from either. One possibility is that individuals are less familiar with constructs related to health advocacy, making it more difficult for them to differentiate across different levels of performance within this competency.

Relatedly, there was a significant proportion of "Not Assessable" responses for items pertaining to the health advocacy competency for both patient and self-assessment versions of the questionnaire. The high proportion of "Not Assessable" responses can be problematic from a validity evidence perspective, as it suggests that such items may not be relevant in the intended clinical context. Whether to remove these items from the questionnaire ultimately depends on why they were marked as "Not Assessable." Is it that the health advocacy behaviors assessed using the present questionnaire are not regularly applicable in these physician-patient interactions? For example, one item refers to whether the physician offered referrals to other support services (e.g., social worker, psychologist, etc.). If few patients require such referrals, then the rating of "Not Assessable" is appropriate, and it may be worthwhile to remove the item. Alternately, it may be that certain health advocacy behaviors should be addressed in these consultations but are simply not being done. In these instances, the rating of "Not Assessable" may be selected because the physician did not do the activity (rather than doing it poorly). This is an important distinction—was the "Not Assessable" rating provided because the behavior was not applicable to the context and therefore could not be assessed, or was the rating provided because the behavior was not observed, even though it was applicable to the context? If the latter instance is true, then the "Not Assessable" responses provide important feedback to educators. Unfortunately, the present study was not able to address this question. Future research should consider a mixed-methods approach to investigate why specific health advocacy items are rated as "Not Assessable."

Furthermore, our study found that self-assessment scores were significantly lower than patient scores across all competencies. This reflects a well-documented propensity for patients to rate their physicians more favourably.[12,27-29] This trend is particularly prevalent when patients have established a relationship with their physicians and are in good health.[29] While the accuracy of self-assessment measures has been highly criticized in the literature,[15] collecting self-assessment data along with patient assessments may provide unique opportunities for self-reflection and self-directed learning.

Strengths and Limitations

Most studies reporting on self-assessment and MSF have compared learner self-assessment to faculty assessment, and there have been mixed findings on the concordance of these two measures.[11,21,28,33-36] A great strength of our work is the unique investigation of trainee self-assessment with patients as the primary comparators. Our findings would have been strengthened by triangulating the self-assessment and patient-assessment scores with faculty feedback, as previously implemented by several groups.[8,28,33] Meaningful faculty feedback serves as an important third source to improve trainee self-reflection and likely accelerate skill acquisition. Since our questionnaire data were collected following initial consultations, we avoided the bias to rate clinicians favorably based on familiarity and trust.[29]

One major limitation was our small learner sample size, which hindered our ability to study level-of-training differences.[29] In the field of medical education, tools that can discriminate between learners by level of study lend evidence to extrapolation validity.[25] While beta testing of our preliminary tool would have strengthened scoring validity, this was not possible in the current study. Additionally, our small sample size limited our evidence for Kane's generalization inference. Our data were confined to a sample of patients and trainees from a single center, potentially limiting generalization. Our findings were also subject to self-selection bias given that enrollment was voluntary. We also identified our rating scale as another possible limitation. Options ranged from excellent to poor on our 5-point semantic-coded scale. We recognize that the tendency for patients to rate their

Copyright © 2021 Esteves et al. This is an open-access publication distributed under the terms of the Creative Commons Attribution license.

physicians leniently could have been partially mitigated by the use of behavioral anchors. Behavioral anchors could help to avoid concerns of health literacy and to standardize patient scoring as they would add vignettes to describe poor to excellent behavior.

Future Directions

Our results demonstrate that the OCCAT produces reliable, validated scores within our population. We plan to implement the questionnaire in conjunction with shared patient feedback as a standard assessment tool for the studied CanMEDS roles in our program. Several items in the OCCAT are aligned with recent EPAs[22] drafted by Canadian NPM programs, and we anticipate that this will facilitate bridging to the competency-based medical education model. Outside our institution, we anticipate that the OCCAT will be utilized by NPM and MFM fellowship programs that are also informed by the CanMEDS 2015 framework and transitioning to competency-based medical education. The OCCAT may serve as a prototype that can be tailored and developed for use by training programs outside the disciplines of NPM and MFM. While some items, especially those pertaining to counseling, are specific to the fields of MFM and NPM, they can be easily adapted to other disciplines.

From this investigation, we recommend several ways to improve the OCCAT for use in future studies. To begin, we recommend studying the OCCAT in a larger sample size and conducting beta testing if resources permit. We have considered the addition of behavioral anchors to the rating scale to improve comprehensibility; however, this would significantly increase the length of the tool. Alternatively, we recommend changing the scale descriptor *poor* to *unsatisfactory* to improve comprehension and allow for fluid comparison to the other descriptors, *less than satisfactory, satisfactory, good,* and *excellent.* More subtly, we recommend presenting the descriptors for the last two items in the column headers as informed by the split-attention principle in cognitive load theory. Integrating these lessons, we have provided readers with the most polished version of the OCCAT, presented as Version 1.1 (Appendix B), for further research and application. Apart from these changes, minor revisions to formatting and spelling have been made. In Version 1.1, we have opted to avoid acronyms such as NICU, and we recommend that institution-specific acronyms be avoided in future use.

Conclusion

Medical education is moving away from time-based requirements to competency-based requirements.[21] It is widely recognized across the medical education community that there is a lack of reliable, robust methods to assess physician learners on non–medical expert competencies.[2,4] We found support for the formal use of the OCCAT using Kane's validity framework[18] and through positive patient and learner feedback.

Patient feedback is an important component of MSF that is recognized as a legitimate and useful tool for assessing the CanMEDS competencies[9] and is indicated to prompt positive changes in practice.[10,11] Patients are invaluable stakeholders in physician education, offering the largest role in assessment of humanistic skills.[33]

Furthermore, self-assessment is vital for professional development.[37] Not only does self-assessment inspire personal reflection, it also serves as a primer for formal evaluations with faculty, allows evaluators to provide useful constructive criticism, and shifts learner motivation from extrinsic to intrinsic.[35,38] Our findings support the integration of self-assessment measures with patient feedback in formal evaluations in order to enrich learner experiences and improve patient outcomes. We hope that the OCCAT will further contribute to competency-based medical education strategies by allowing trainees and faculty to fill the gap of nonobserved performances, with the aim of promoting an additional step towards transition to practice.

Appendices

A. Patient Recruitment.docx

B. OCCAT Version 1.1.docx

All appendices are peer reviewed as integral parts of the Original Publication.

Ashley Esteves, BScH: Senior Medical Student, University of Ottawa Faculty of Medicine

Meghan McConnell, PhD: Associate Professor, Department of Innovation in Medical Education and Department of Anesthesiology and Pain Medicine, University of Ottawa Faculty of Medicine

Emanuela Ferretti, MD, MSc: Neonatologist and Associate Professor, Division of Neonatology, Department of Pediatrics, Children's Hospital of Eastern Ontario and University of Ottawa Faculty of Medicine

Adam Garber, MD, MSc: Associate Program Director and Associate Professor, Department of Obstetrics and Gynecology, University of Ottawa Faculty of Medicine

Copyright © 2021 Esteves et al. This is an open-access publication distributed under the terms of the Creative Commons Attribution license.

Karen Fung-Kee-Fung, MD, MHPE: Professor, Division of Maternal Fetal Medicine, Department of Obstetrics and Gynecology, University of Ottawa Faculty of Medicine

Acknowledgments
We would foremost like to thank the patients for their participation in making this study possible. We would like to acknowledge our dedicated neonatal-perinatal medicine and maternal fetal medicine fellows MS, GM, HH, SL, MC, MG, ZP, AA, and GH for striving to become better doctors and helping others to do the same. We would also like to acknowledge the Post Graduate Medical Education office at the University of Ottawa for financial support of this project through the initiatives for residency education program.

Disclosures
None to report.

Funding/Support
None to report.

Ethical Approval
The Ottawa Health Science Network Research Ethics Board approved this study.

References

1. Frank JR, Snell L, Sherbino J, eds. *CanMEDS 2015 Physician Competency Framework*. Royal College of Physicians and Surgeons of Canada; 2015.
2. Whitehead CR, Kuper A, Hodges B, Ellaway R. Conceptual and practical challenges in the assessment of physician competencies. *Med Teach*. 2015;37(3):245-251. https://doi.org/10.3109/0142159X.2014.993599
3. Holmboe ES, Sherbino J, Long DM, Swing SR, Frank JR; International CBME Collaborators. The role of assessment in competency-based medical education. *Med Teach*. 2010;32(8): 676-682. https://doi.org/10.3109/0142159X.2010.500704
4. Chou S, Cole G, McLaughlin K, Lockyer J. CanMEDS evaluation in Canadian postgraduate training programmes: tools used and programme director satisfaction. *Med Educ*. 2008;42(9):879-886. https://doi.org/10.1111/j.1365-2923.2008.03111.x
5. Warren AE, Allen VM, Bergin F, et al. Understanding, teaching and assessing the elements of the CanMEDS professional role: Canadian program directors' views. *Med Teach*. 2014;36(5): 390-402. https://doi.org/10.3109/0142159X.2014.890281
6. Roberts GG, Bieko D, Touma N, Siemens R. Are we getting through? A national survey on the CanMEDS communicator role in urology residency. *Can Urol Assoc J*. 2013;7(11-12):437-441. https://doi.org/10.5489/cuaj.264
7. Puddester D, MacDonald CJ, Clements D, Gaffney J, Wiesenfeld L. Designing faculty development to support the evaluation of resident competency in the intrinsic CanMEDS roles: practical outcomes of an assessment of program director needs. *BMC Med Educ*. 2015;15:100. https://doi.org/10.1186/s12909-015-0375-5
8. Chandler N, Henderson G, Park B, Byerley J, Brown WD, Steiner MJ. Use of a 360-degree evaluation in the outpatient setting: the usefulness of nurse, faculty, patient/family, and resident self-evaluation. *J Grad Med Educ*. 2010;2(3):430-434. https://doi.org/10.4300/JGME-D-10-00013.1
9. Lockyer J. Multi-source feedback (360-degree evaluation). In: Bandiera G, Sherbino J, Frank JR, eds. *The CANMEDS Assessment Tools Handbook: An Introductory Guide to Assessment Methods for the CanMEDS Competencies*. Royal College of Physicians and Surgeons of Canada; 2006:29-31.
10. Lockyer J, Armson H, Chesluk B, et al. Feedback data sources that inform physician self-assessment. *Med Teach*. 2011;33(2):e113-e120. https://doi.org/10.3109/0142159X.2011.542519
11. Brinkman WB, Geraghty SR, Lanphear BP, et al. Effect of multisource feedback on resident communication skills and professionalism: a randomized controlled trial. *Arch Pediatr Adolesc Med*. 2007;161(1):44-49. https://doi.org/10.1001/archpedi.161.1.44
12. Donnon T, Al Ansari A, Al Alawi S, Violato C. The reliability, validity, and feasibility of multisource feedback physician assessment: a systematic review. *Acad Med*. 2014;89(3): 511-516. https://doi.org/10.1097/ACM.0000000000000147
13. Bandiera G, Sherbino J, Frank JR, eds. *The CanMEDS Assessment Tools Handbook: An Introductory Guide to Assessment Methods for the CanMEDS Competencies*. Royal College of Physicians and Surgeons of Canada; 2006.
14. Epstein RM, Siegel DJ, Silberman J. Self-monitoring in clinical practice: a challenge for medical educators. *J Contin Educ Health Prof*. 2008;28(1):5-13. https://doi.org/10.1002/chp.149
15. Eva KW, Regehr G. Self-assessment in the health professions: a reformulation and research agenda. *Acad Med*. 2005;80(suppl 10):S46-S54.
16. Sharma R, Jain A, Gupta N, Garg S, Batta M, Dhir SK. Impact of self-assessment by students on their learning. *Int J Appl Basic Med Res*. 2016;6(3):226-229. https://doi.org/10.4103/2229-516X.186961
17. Bourke R. Liberating the learner through self-assessment. *Cambridge J Educ*. 2016;46(1):97-111. https://doi.org/10.1080/0305764X.2015.1015963
18. Kane MT. Validating the interpretations and uses of test scores. *J Educ Meas*. 2013;50(1):1-73. https://doi.org/10.1111/jedm.12000
19. Fluit C, Bolhuis S, Grol R, et al. Evaluation and feedback for effective clinical teaching in postgraduate medical education: validation of an assessment instrument incorporating the CanMEDS roles. *Med Teach*. 2012;34(11):893-901. https://doi.org/10.3109/0142159X.2012.699114

Copyright © 2021 Esteves et al. This is an open-access publication distributed under the terms of the Creative Commons Attribution license.

20. Nation JG, Carmichael E, Fidler H, Violato C. The development of an instrument to assess clinical teaching with linkage to CanMEDS roles: a psychometric analysis. *Med Teach*. 2011;33(6):e290-e296. https://doi.org/10.3109/0142159X.2011.565825

21. Probyn L, Lang C, Tomlinson G, Bandiera G. Multisource feedback and self-assessment of the communicator, collaborator, and professional CanMEDS roles for diagnostic radiology residents. *Can Assoc Radiol J*. 2014;65(4):379-384. https://doi.org/10.1016/j.carj.2014.04.003

22. *Entrustable Professional Activities for Neonatal-Perinatal Medicine*. Royal College of Physicians and Surgeons of Canada; 2020. Accessed May 25, 2021. https://www.royalcollege.ca/rcsite/documents/cbd/neonatal-perinatal-medicine-epas-e.pdf

23. Downing SM. Validity: on the meaningful interpretation of assessment data. *Med Educ*. 2003;37(9):830-837. https://doi.org/10.1046/j.1365-2923.2003.01594.x

24. Cook DA, Brydges R, Ginsburg S, Hatala R. A contemporary approach to validity arguments: a practical guide to Kane's framework. *Med Educ*. 2015;49(6):560-575. https://doi.org/10.1111/medu.12678

25. Cook DA, Hatala R. Validation of educational assessments: a primer for simulation and beyond. *Adv Simul (Lond)*. 2016;1:31. https://doi.org/10.1186/s41077-016-0033-y

26. Cook DA, Beckman TJ. Current concepts in validity and reliability for psychometric instruments: theory and application. *Am J Med*. 2006;119(2):166.E7-166.E16. https://doi.org/10.1016/j.amjmed.2005.10.036

27. Archer JC, McAvoy P. Factors that might undermine the validity of patient and multi-source feedback. *Med Educ*. 2011;45(9):886-893. https://doi.org/10.1111/j.1365-2923.2011.04023.x

28. Keister DM, Hansen SE, Dostal J. Teaching resident self-assessment through triangulation of faculty and patient feedback. *Teach Learn Med*. 2017;29(1):25-30. https://doi.org/10.1080/10401334.2016.1246249

29. Lipner RS, Blank LL, Leas BF, Fortna GS. The value of patient and peer ratings in recertification. *Acad Med*. 2002;77(10)(suppl):S64-S66. https://doi.org/10.1097/00001888-200210001-00021

30. Cohen J. *Statistical Power Analysis for the Behavioral Sciences*. Academic Press; 2013.

31. Bland JM, Altman DG. Statistics notes: Cronbach's alpha. *BMJ*. 1997;314(7080):572. https://doi.org/10.1136/bmj.314.7080.572

32. Tavakol M, Dennick R. Making sense of Cronbach's alpha. *Int J Med Educ*. 2011;2:53-55. https://doi.org/10.5116/ijme.4dfb.8dfd

33. Wood J, Collins J, Burnside ES, et al. Patient, faculty, and self-assessment of radiology resident performance: a 360-degree method of measuring professionalism and interpersonal/communication skills. *Acad Radiol*. 2004;11(8):931-939. https://doi.org/10.1016/j.acra.2004.04.016

34. Ross FJ, Metro DG, Beaman ST, et al. A first look at the Accreditation Council for Graduate Medical Education anesthesiology milestones: implementation of self-evaluation in a large residency program. *J Clin Anesth*. 2016;32:17-24. https://doi.org/10.1016/j.jclinane.2015.12.026

35. Meier AH, Gruessner A, Cooney RN. Using the ACGME milestones for resident self-evaluation and faculty engagement. *J Surg Educ*. 2016;73(6):e150-e157. https://doi.org/10.1016/j.jsurg.2016.09.001

36. Moroz A, Horlick M, Mandalaywala N, Stern DT. Faculty feedback that begins with resident self-assessment: motivation is the key to success. *Med Educ*. 2018;52(3):314-323. https://doi.org/10.1111/medu.13484

37. Ganni S, Botden SMBI, Schaap DP, Verhoeven BH, Goossens RHM, Jakimowicz JJ. "Reflection-*before*-practice" improves self-assessment and end-performance in laparoscopic surgical skills training. *J Surg Educ*. 2018;75(2):527-533. https://doi.org/10.1016/j.jsurg.2017.07.030

38. Tekian A, Watling CJ, Roberts TE, Steinert Y, Norcini J. Qualitative and quantitative feedback in the context of competency-based education. *Med Teach*. 2017;39(12):1245-1249. https://doi.org/10.1080/0142159X.2017.1372564

Received: August 16, 2020
Accepted: May 7, 2021
Published: July 21, 2021

Copyright © 2021 Esteves et al. This is an open-access publication distributed under the terms of the Creative Commons Attribution license.
